# Periodontitis as a promoting factor of T2D: current evidence and mechanisms

**DOI:** 10.1038/s41368-023-00227-2

**Published:** 2023-06-15

**Authors:** Yuan Su, Leilei Ye, Chen Hu, Yanli Zhang, Jia Liu, Longquan Shao

**Affiliations:** 1 Stomatology Center, Shunde Hospital, Southern Medical University (The First People’s Hospital of Shunde), Foshan, China; 2 Stomatological Hospital, School of Stomatology, Southern Medical University, Guangzhou, China; 3 Department of Stomatology, Nanfang Hospital, Southern Medical University, Guangzhou, China

**Keywords:** Type 2 diabetes, Inflammatory diseases, Periodontitis, Bacterial infection, Mechanisms of disease

## Abstract

Periodontitis is an infectious disease caused by an imbalance between the local microbiota and host immune response. Epidemiologically, periodontitis is closely related to the occurrence, development, and poor prognosis of T2D and is recognized as a potential risk factor for T2D. In recent years, increasing attention has been given to the role of the virulence factors produced by disorders of the subgingival microbiota in the pathological mechanism of T2D, including islet β-cell dysfunction and insulin resistance (IR). However, the related mechanisms have not been well summarized. This review highlights periodontitis-derived virulence factors, reviews how these stimuli directly or indirectly regulate islet β-cell dysfunction. The mechanisms by which IR is induced in insulin-targeting tissues (the liver, visceral adipose tissue, and skeletal muscle) are explained, clarifying the influence of periodontitis on the occurrence and development of T2D. In addition, the positive effects of periodontal therapy on T2D are overviewed. Finally, the limitations and prospects of the current research are discussed. In summary, periodontitis is worthy of attention as a promoting factor of T2D. Understanding on the effect of disseminated periodontitis-derived virulence factors on the T2D-related tissues and cells may provide new treatment options for reducing the risk of T2D associated with periodontitis.

## Introduction

Type 2 diabetes (T2D), a chronic metabolic disease, is characterized by pancreatic β-cell dysfunction and decreased metabolic responses of peripheral insulin-targeting tissue, such as the liver, visceral adipose tissue, and skeletal muscle, when insulin sensitivity is impaired, namely, during insulin resistance (IR). T2D has become a public health problem worldwide. According to data published by the International Diabetes Federation, the number of individuals with diabetes worldwide was ~460 million in 2019 and will reach 700 million by 2045, and most of these individuals are T2D patients. The global healthcare cost of diabetes is $760 billion per year, and this value is expected to increase to $845 billion per year by 2045.^1^ From Western countries to the Western Pacific, Asia, and Africa, premature death, disability, and other complications caused by T2D have led to adverse economic and social impacts.

**Fig. 1:**
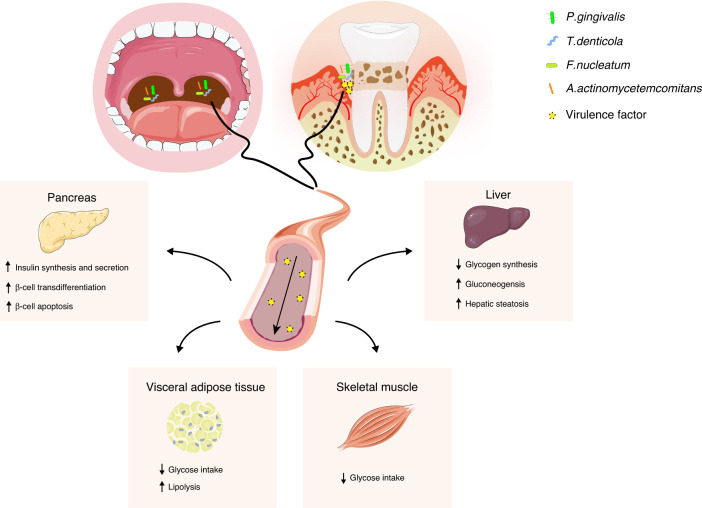
Mechanism by which periodontitis promotes T2D occurrence and development. Periodontitis-derived virulence factors can enter blood circulation through the deep periodontal pocket or through the gastrointestinal tract and lead to β-cell dysfunction in the pancreas and abnormal glucose and lipid metabolism in peripheral insulin-targeting tissues, such as the liver, visceral adipose tissue, and skeletal muscle

It has been confirmed that T2D and periodontitis can interact with each other.^2^ T2D exacerbates periodontitis by enhancing local inflammatory reactions in periodontal tissues or by disrupting the homeostasis of subgingival microorganisms.^3–5^ However, this review focuses on how periodontitis affects the occurrence and development of T2D. The etiology of T2D is complex, and it includes a variety of genetic factors and acquired risk factors. Increasing evidence shows that after adjusting for known metabolic risk factors, periodontitis has emerged as another important cause of the occurrence and development of T2D.^6,7^ Periodontitis is an infectious disease caused by an imbalance between the local microbiota and host immune response. Microbial dysbiosis in periodontitis leads to an increase in periodontal pathogens and the production of a large number of toxic products, which excessively activate the host’s immune response and mediate periodontal tissue damage.^8^ As the sixth pandemic that affects humankind, periodontitis affects 20–50% of the global population;^9^ the worldwide prevalence of severe periodontitis is as high as 10.8%, and it affects 743 million people.^10^ In recent years, periodontitis has attracted increasing attention as a factor that promotes T2D occurrence and development. There is a close relationship between the virulence factors that are produced as a result of microbial dysbiosis in periodontitis and the pathological changes that occur during T2D. However, the relevant pathological mechanisms underlying these connections have not yet been discussed and reviewed. Therefore, this review highlights the virulence factors produced by disorders of the subgingival microbiota and reviews how these stimuli directly or indirectly regulate islet β-cell dysfunction. The mechanisms by which IR is induced in insulin-targeting tissues (the liver, visceral adipose tissue, and skeletal muscle) are explained (Fig. 1), clarifying the influence of periodontitis on the occurrence and development of T2D and providing more specific recommendations and strategies for the clinical prevention and control of T2D.

## Periodontitis as a promoting factor in T2D

### Clinical evidence

Extensive clinical evidence has indicated that periodontitis affects the occurrence, progression, and prognosis of T2D (Table 1). Systemically healthy subjects with periodontitis who do not receive standard periodontal treatment may present with an extended range of inflammation that affects individual teeth to the full mouth. Periodontal probing depth (PPD) and attachment loss also increase. Fasting blood glucose (FBG) levels in these patients tend to increase to levels near the value for diagnosis of prediabetes,^11^ and then, these patients exhibit abnormal glucose metabolism, such as impaired fasting glycemia (IFG)^12^ and impaired glucose tolerance (IGT),^13^ before the onset of T2D. Prospective cohort studies have shown that untreated patients with periodontitis can also present with decreased serum levels of glucagon-like peptide-1 (GLP-1) compared with healthy periodontal controls.^14^ Periodontitis patients exhibited a 33% increased risk of hyperglycemia^15^ and a 1.24-fold increased risk of developing T2D^16^ after >5 years of follow-up. In addition, patients with T2D and periodontitis showed significantly higher FBG levels, increased homeostasis model assessments of IR (HOMA-IR) levels,^11,17^ and decreased homeostasis model assessment of β-cell function (HOMA-β) levels,^18^ suggesting that periodontitis exacerbates β-cell dysfunction and peripheral IR in T2D patients. In terms of prognosis, periodontitis can increase the HbA1c level,^19^ which is the prognostic target for T2D, and increase the risk of diabetic nephropathy (OR: 1.55, 95% CI: 1.24–1.94),^20^ diabetic retinopathy (OR: 1.193, 95% CI: 0.757–1.881),^21^ diabetic microangiopathy (OR: 2.43, 95% CI: 1.65–3.56),^22^ and other complications.

**Table 1:** Clinical evidence of periodontitis as a promoting factor of T2D

Design	Sample size and characteristics	Exposure	Main outcomes
Cross-sectional study^11^	37 patients with severe periodontitis and 37 patients with healthy periodontium.	•PPD •CAL	FBG (*P* < 0.001), HbA1c (*P* < 0.05), HOMA-IR (*P* < 0.05), and IFG (OR:7.489, 95% CI: 1.408–39.839; *P* < 0.01) were increased in patients with severe periodontitis.
Cross-sectional study^12^	5070 patients with periodontitis and 12,108 patients without periodontitis	•CPI score	IFG (*P* < 0.001) was increased and HOMA-β (*P* < 0.001) was decreased in periodontitis patients.
Cross-sectional study^13^	665 patients with no/mild periodontitis and 500 patients with moderate/severe periodontitis	•PPD •CAL	IGT was increased in patients with moderate (OR:1.07, 95% CI: 0.50-2.25; *P* = 0.02) or severe (OR:1.93, 95% CI:1.18–3.17; *P* = 0.02) periodontitis.
Cross-sectional study^14^	47 patients with periodontitis and 63 patients without periodontitis among severely obese, nondiabetic individuals	•PPD •BOP •CAL	The serum GLP-1 level (*P* < 0.000 1) was decreased in periodontitis patients.
Prospective cohort study, Follow-up:5 years^15^	1341 patients with periodontitis and 4033 patients without periodontitis	•CPI score	Incidence of hyperglycemia was increased by 33% (aHR:1.33, 95% CI :1.09–1.63) in periodontitis patients.
Retrospective cohort study, Mean follow-up: 5.47 ± 3.54 years^16^	22299 patients with periodontitis needing dental surgery and 22302 patients with periodontitis not requiring dental surgery	•Subgingival curettage •Periodontal flap procedure	Incidence of T2D was 1.24-fold higher (aHR:1.19,95% CI :1.10–1.29) in patients with periodontitis needing dental surgery.
Cross sectional study^17^	207 patients with periodontitis and 67 patients without periodontitis in T2D patients	•PPD •CAL •LGM •PI •GI	FBG (*P* < 0.000 1), HbA1c (*P* < 0.000 1) and the risk of diabetes-associated complications (*P* < 0.000 1) were increased in T2D patients with periodontitis patients.
Cross sectional study^18^	20 T2D patients with periodontitis, 20 T2D patients without periodontitis and 20 nondiabetic controls with periodontitis	•BOP •PPD	HbA1c (*P* = 0.002) and FBG (*P* = 0.04) were increased and HOMA-β (*P* = 0.01) was decreased in T2D with periodontitis patients.
Cross sectional study^19^	50 patients with chronic periodontitis and 50 patients without periodontitis.	•PI •OHI-s •MGI •PPD •CAL	HbA1c (*P* = 0.001) and FBG (*P* = 0.002) were increased in chronic periodontitis patients.
Cross sectional study^24^	126 patients with none/mild periodontitis, 156 patients with moderate periodontitis and 18 patients with severe periodontitis	•Bacterial burden score	Higher colonization levels of specific periodontal microbiota are associated with higher prediabetes prevalence among diabetes-free adults (*A. actinomycetemcomitans*, 2.48 (1.34, 4.58), *P* = 0.004; *P. gingivalis*, 3.41 (1.78, 6.58), *P* = 0.000 3; *T. denticola*, 1.99 (0.992, 4.00), *P* = 0.052).
Cross sectional study^25^	114 patients with none/mild periodontitis, 159 patients with moderate periodontitis and 27 patients with severe periodontitis.	•The relative abundance of bacterial phyla	The levels of *Actinobacteria*, *Proteobacteria* or *Firmicutes* were associated with insulin resistance (*P* < 0.05), and the strongest positive correlations between bacterial taxa and HOMA-IR was for *Prevotella* sp. HOT-299_AH07, *r* = 0.21 (*P* = 0.01).
Cross sectional study^26^	52 non-alcoholic fatty liver disease	•IgG antibody titers	Anti-*A. actinomycetemcomitans* IgG antibody titer correlated positively with the HOMA-IR (*P* = 0.001, *ρ* = 0.46).
Cross sectional study^7^	97 patients with none/mild periodontitis, 117 patients with moderate periodontitis and 16 patients with severe periodontitis.	•Microbial dysbiosis index	Higher levels of the microbial dysbiosis index were strongly associated with higher levels of glucose change after multivariable adjustment (*P* < 0.000 1). Baseline levels of 9 taxa predicted FBG change (all FDR < 0.05), among which *Stomatobaculum* sp oral taxon 097 and *Atopobium* spp predicted greater FBG change.

*T2D* type 2 diabetes, *PPD* probing pocket depth, *CAL* clinical attachment level, FBG fasting blood glucose, *IFG* impaired fasting glucose, *OR* odds ratio, CI confidence intervals, *aHR* adjusted hazard ratio, *HbA1c* glycated hemoglobin, *HOMA-IR* homeostasis model assessments of IR, *CPI* community periodontal index, *HOMA-β* homeostasis model assessments of β-cell function, *IGT* impaired glucose tolerance, *BOP* bleeding on probing, *GLP-1* glucagon-like peptide-1, *LGM* location of gingival margin, *PI* plaque index, *GI* gingival index, *OHI-s* simplified oral hygiene index, *MGI* modified gingival index, *FDR* false discovery rate

In fact, the changes in the periodontal clinical parameters mentioned above are indirect reflections of subgingival microbial dysbiosis.^23^ The measurement and analysis of these clinical parameters are relatively simple, so they are often used to replace the analysis of the subgingival microbiota in the study of the effect of periodontitis on T2D. In recent years, with the development and use of metagenomic next-generation sequencing, direct evidence has been reported that subgingival microbial dysbiosis is related to the occurrence and development of T2D. Studies have confirmed that the increased abundance of *P. gingivalis* (OR: 3.41, 95% CI: 1.78–6.58), *A. actinomycetemcomitans* (OR: 2.48, 95% CI: 1.34–4.58), *F. nucleatum* (OR: 2.43, 95% CI: 1.32–4.48), and *T. denticola* (OR: 1.99, 95% CI: 0.992–4.00), which is caused by subgingival microbial dysbiosis, is related to the increase in FBG, HbA1c^24^ and HOMA-IR levels^25,26^ and increases in the risk of T2D. Moreover, in young individuals with systematic health, changes in subgingival microbial abundance are an important indicator that is better than age and body mass index (BMI) for predicting the future occurrence of T2D.^7^

### Periodontitis-derived virulence factors

Fundamentally, the increased abundance of *P. gingivalis*, *A. actinomycetemcomitans*, *F. nucleatum* and *T. denticola* caused by microbial dysbiosis in periodontitis leads to excessive levels of virulence factors,^8^ including lipopolysaccharide (LPS) secreted by *P. gingivalis* and *F. nucleatum*, gingipain and outer membrane vesicles (OMVs) from *P. gingivalis*, and leukotoxin secreted by *A. actinomycetemcomitans*. These virulence factors can directly interact with T2D-related target cells^27–29^ or interfere with target cell function through indirect immune regulation,^30,31^ thus affecting the occurrence, development and prognosis of T2D.

Studies have confirmed that LPS is the main component of the gram-negative bacterial cell wall.^32^ LPS can induce specific immune modulation through Toll-like receptors (TLRs) in T2D-related target cells, such as β-cells, hepatocytes, and visceral adipocytes, and activate the inflammatory signaling pathway, leading to β-cell dysfunction and IR.^33–35^ Gingipain and OMVs are also key virulence factors that induce IR. Gingipain, which is a cysteine protease family member, can be synthesized and later released into the extracellular environment. Gingipain enhances virulence by inducing the degradation of extracellular matrix proteins, cleavage of cell receptors, degradation of fibrinogen, and inactivation of complement system components.^36^ OMVs, which have spherical nanostructures with a membrane bilayer and a diameter of 50 ~ 250 nm,^37^ contain various virulence factors and exhibit greater immunogenicity and invasive ability.^38^ Therefore, they can invade tissue cells far from the oral cavity, induce metabolic abnormalities^28^ and exacerbate IR.

In terms of indirect immune regulation, leukotoxins can cause dysfunction of neutrophils and monocytes,^39,40^ disrupt the innate immune response of the body, and participate in IR. LPS can enter the systemic circulation, activate the innate immune response, and stimulate macrophages and dendritic cells to secrete a large number of inflammatory factors^41^ that cause endotoxemia and chronic inflammation of the body and induce IR.^42^ Moreover, LPS can further lead to local and systemic adaptive immune response disorders, damage glucose metabolism and promote IR.^30^

In addition to the aforementioned classical virulence factors, other periodontitis-derived virulence factors have been observed in T2D-related target tissues. These virulence factors include the pili protein FimA, dipeptidyl peptidase (DPP), prolyl tripeptidyl peptidase (PTP), and the bacterial elongation factor Tu (EF-Tu).^28^ Among these factors, FimA may competitively inhibit the binding of angiopoietin 2 to α5β1-integrin,^43^ leading to ectopic lipid deposition and further IR.^44^ *P. gingivalis*-derived DPP4 and intestinal-derived DPP4 share 31% homology. *P. gingivalis*-derived DPP4 may bind to PTP^45^ and then participate in the degradation of GLP-1 in circulation and inhibit insulin secretion from β-cells.^46^ Similar to LPS, EF-Tu, which is a pathogen-associated molecular pattern protein, can activate the immune-inflammatory response of host cells^47^ and thus contribute to IR. Further work is needed to identify the influence of these virulence factors on the pathological mechanism underlying T2D.

Currently, it is believed that periodontitis-derived virulence factors can enter the blood circulation through an ulcerated surface in the periodontal pocket^48–51^ or through saliva to the gastrointestinal tract^52^ and travel to the pancreas or other insulin-targeting tissues.^27–29,53^ Of those, β-cells in the pancreas have a high affinity for these virulence factors.^27^ After long-term stimulation with virulence factors, β-cell status shifts from a compensatory state to a decompensatory state, thereby becoming dysfunctional.

## Mechanism underlying β-cell dysfunction regulation

The compensatory responses of β-cells manifest primarily as increased insulin synthesis and secretion in response to the metabolic demands induced by stress stimuli to stabilize blood glucose. As stimulation increases, β-cells undergo decompensation with progressive losses of mass, function and insulin-secreting capacity, leading to T2D. Studies have shown that many individuals with severe periodontitis that lasts a long time can develop hyperinsulinemia,^27^ which is a compensatory β-cell response. As periodontitis is exacerbated, β-cells undergo transdifferentiation^27^ or apoptosis^53^ and gradually decompensation, which results in decreased insulin secretion and increased FBG levels.

### β-cell compensation

**Fig. 2:**
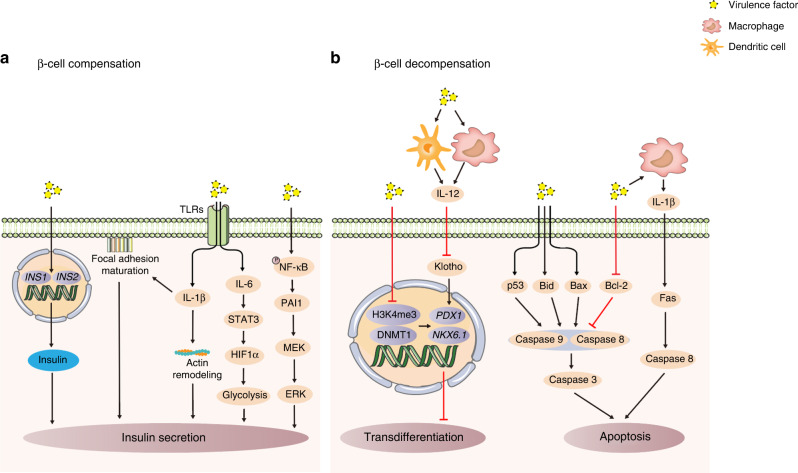
Mechanism underlying β-cell dysfunction induced by periodontitis-derived virulence factors. **a** β-cell compensation. Virulence factors promote insulin synthesis and secretion. **b** β-cell decompensation. Virulence factors induce β-cell transdifferentiation or apoptosis, leading to β-cell decompensation. Black arrows represent activating events; red arrows represent inhibitory events. *INS1* insulin gene 1, *INS2* insulin gene 2, TLRs Toll-like receptors, IL-1β interleukin-1β, IL-6 interleukin-6, STAT3 signal transducer and activator of transcription 3, HIF1α hypoxia inducible factor 1α, NF-κB nuclear factor κB, PAI1 plasminogen activator inhibitor-1, MEK MAP kinse-ERK kinase, ERK extracellular regulated MAP kinase, IL-12 interleukin-12, H3K4me3 histone 3 trimethylated at lysine 4, DNMT1 DNA methyltransferase 1, *PDX1* pancreatic and duodenal homeobox 1, *NKX6.1* NK6 homeobox 1, Bid BH3 interacting domain death agonist, Bax Bcl-2 associated X

Ramenzoni LL et al.^54^ used the supernatant of mixed cultures of *P. gingivalis* and *T. denticola* to simulate the synergistic effect of multiple virulence factors on β-cells after periodontal microbial dysbiosis. The supernatant could promote insulin synthesis and secretion by β-cells (Fig. 2a). On the one hand, stimulation with the supernatant could upregulate the transcription level of the insulin genes *INS1* and *INS2*^54^ and promote insulin synthesis. The transcription of *INS1* and *INS2* is regulated by β-cell-specific transcription factors pancreatic and duodenal homeobox 1 (*PDX1*), MAF bZIP transcription factor A (MafA) and neuronal differentiation 1 (Neurod1). They bind to the A3, C1 and E1 cis-acting DNA elements proximal to the insulin promoter to activate transcription.^55^ However, the mechanism by which periodontitis-derived virulence factors regulate the above transcription factors to initiate insulin gene transcription remains unclear and is worthy of further discussion in the future. On the other hand, this supernatant could activate immune signals in β-cells to promote insulin secretion. TLR receptors on the surface of β-cells were activated by this supernatant, which led to the secretion of the inflammatory cytokines interleukin-1β (IL-1β) and interleukin-6 (IL-6).^33,54^ Among these cytokines, the former promotes the movement of insulin particles to the cell membrane to promote secretion by facilitating the maturation of adhesion plaques and actin remodeling in β-cells.^56^ Additionally, IL-6 could regulate signal transducer and activator of transcription 3-hypoxia inducible factor 1α (STAT3-HIF1α) signaling to enhance glycolysis in β-cells and promote insulin granule secretion.^57^ Unfortunately, the virulence factors in the supernatants of this bacterial coculture have not been identified in previous studies. Virulence factors such as LPS,^33^ and gingipain from *P. gingivalis*,^58^ major surface proteins,^59^ and dentilisin^60^ from *T. denticola* have been reported to have the potential to regulate gene transcription and interfere with immune regulation. Therefore, it is necessary to identify the virulence factors in this supernatant in future studies and to perform an in-depth analysis of how these virulence factors act synergistically to mediate the compensatory response of β-cells.

In the in-depth study of how purified periodontitis-derived virulence factors affect β-cell compensation, LPS has attracted the most attention. This is because LPS is a major participant in periodontitis-induced endotoxemia. This chronic endotoxemia leads to β-cell function compensation and hyperinsulinemia. Although LPS cannot regulate the transcription of the insulin gene to affect insulin synthesis,^33^ it can regulate the immuno-inflammatory signaling pathways in β-cells to promote insulin secretion. LPS can upregulate the expression of CD14 in β-cells to activate TLRs, stimulate the expression of the inflammatory factor plasminogen activator inhibitor 1 (PAI1) through the nuclear factor κB (NF-κB) pathway,^33^ and further activate the MAP kinse-ERK kinase/extracellular regulated MAP kinase (MEK/ERK) pathway^61^ in β-cells to promote insulin secretion.^62^

In addition to β-cell function changes, β-cell proliferation is also an important mechanism that promotes insulin secretion. Some studies have shown that periodontitis-derived virulence factors can upregulate the expression of cyclin E and downregulate the expression of the cell cycle inhibitor cyclin-dependent kinase inhibitor 1 (p21),^63^ while cyclin E and p21 are the key factors that regulate the G1/S stage of the cell cycle that promote β-cell proliferation.^64^ It is suggested that exposure to periodontitis-derived virulence factors has the potential to regulate the proliferation of β-cells, which can be explored in future research.

### β-cell decompensation

**Fig. 3:**
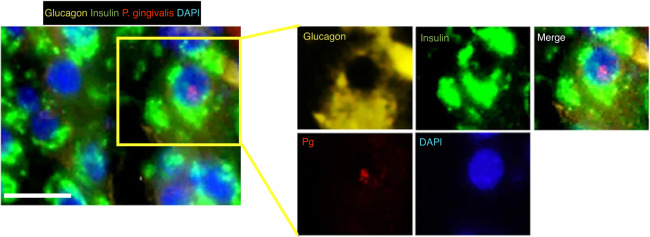
Periodontitis-derived virulence factors induce the generation of bihormonal cells. Bihormonal cells that express both insulin and glucagon were observed in the pancreas of mice with periodontitis, and *P. gingivalis* localization in the nucleus was observed.^27^ with permission

In response to long-term stimulation with periodontitis-derived virulence factors, β-cells in a compensatory state undergo transdifferentiation or apoptosis, gradually losing the ability to secrete insulin and entering the decompensatory state (Fig. 2b). Among these processes, β-cell transdifferentiation means that β-cells transform into cells that express other hormones and lose the ability to secrete insulin. An important hallmark of this process is the appearance of bihormonal cells that express both insulin and another hormone,^65,66^ which is accompanied by a decrease in insulin secretion. It was found that periodontitis-derived virulence factors can selectively localize to β-cells^27^ and induce the emergence of bihormonal cells (Fig. 3) through epigenetic modifications, such as reducing the abundance of histone 3 trimethylated at lysine 4 (H3K4me3)^67^ and decreasing DNA methyltransferase 1 (DNMT1),^68–70^ to downregulate the β-cell-specific transcription factors *PDX1* and NK6 homeobox 1 (*NKX6.1*)^71^ and induce the development of bihormonal cells. It is worth noting that periodontitis-derived virulence factors can target β-cells to promote their transdifferentiation in both human and rodent islets.^27^ This may be an important pathological basis by which periodontitis promotes the occurrence and development of T2D, but the targeting mechanism remains unclear. This may be related to the expression of receptors on the surface of β-cells that can specifically bind to these virulence factors. It is necessary to further study the targeting mechanism in the future, and it is very important to block the effect of periodontitis-derived virulence factors on β-cells and prevent T2D in the early stage.

In addition to directly affecting β-cells, periodontitis-derived virulence factors can indirectly regulate the transdifferentiation of β-cells by regulating the immune response. During periodontitis, LPS can stimulate innate immune cells, such as dendritic cells and macrophages, to secrete a large amount of IL-12,^41^ resulting in a significant increase in serum IL-12 levels.^72^ High levels of IL-12 can downregulate the protein expression of Klotho in β-cells,^73^ inhibit the activity of the transcription factor *PDX1*,^73^ and promote the transdifferentiation of β-cells into α-cells,^74^ thus exacerbating the dysfunction of insulin secretion by β-cells.

A decrease in the number of β-cells due to apoptosis is also another important mechanism leading to decompensation. Periodontitis-derived virulence factors can regulate β-cell apoptosis through the death receptor pathway and mitochondrial pathway.^53^ It has been confirmed that direct stimulation by *P. gingivalis* can activate Caspase 8, which is a key protein in the death receptor pathway, Caspase 9, which is a key protein in the mitochondrial pathway, and downstream Caspase 3, resulting in β-cell apoptosis.^53^ These effects may be related to the gingipain and LPS that are secreted by *P. gingivalis*, which have been proven to upregulate the proapoptotic proteins p53, BH3 interacting domain death agonist (Bid), and Bcl-2 associated X (Bax), downregulate Bcl-2,^75,76^ and activate Caspase 8, Caspase 9 and Caspase 3 to mediate apoptosis.^77^ In addition, periodontitis-derived virulence factors stimulate islet macrophages to secrete large amounts of IL-1β,^78^ which indirectly activates the death receptor Fas and Caspase 8 in β-cells in a paracrine manner, leading to apoptosis.^79^ However, few studies have described direct or indirect regulatory effects of these virulence factors on β-cell apoptosis; the main focus of previous studies has been on the effect of virulence factors on the expression of apoptotic markers downstream of the pathway, while the upstream regulatory mechanisms remain unclear. Further studies should focus on identifying upstream signaling molecules in β-cells that act as targets for periodontitis-derived virulence factors, which will help to elucidate the molecular basis by which periodontitis exacerbates T2D and provide a basis for identifying new drug targets to combat β-cell apoptosis.

In addition to transdifferentiation and apoptosis, another important mechanism of β-cell decompensation is dedifferentiation; that is, β-cells lose endocrine function and degenerate into pancreatic progenitor cells. Studies have confirmed that stimulation of periodontitis-derived virulence factors upregulates the expression of the transcription factor forkhead box O1 (*FOXO1*) in hepatocytes,^80^ which is a key transcription factor that regulates β-cell dedifferentiation.^81^ However, there is no direct evidence to prove that periodontitis-derived virulence factors can regulate β-cell dedifferentiation via *FOXO1* by the same mechanism.

## Mechanism underlying IR regulation

### Liver IR

**Fig. 4:**
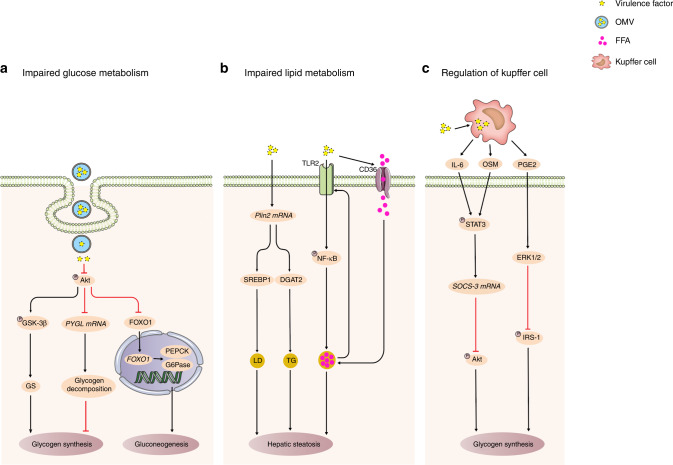
Mechanism underlying liver IR induced by periodontitis-derived virulence factors. **a** Impaired glucose metabolism. Virulence factors can result in abnormal glucose metabolism and participate in liver IR through decreased glycogen synthesis and increased gluconeogenesis. **b** Impaired lipid metabolism. Virulence factors can result in steatosis through ectopic deposition of FFAs, contributing to liver IR. **c** Regulation of Kupffer cell. Virulence factors can accumulate in Kupffer cells and activate them. Activated Kupffer cells can secrete IL-6, OSM and PGE2, leading to hepatocyte IR. Black arrows represent activating events; red arrows represent inhibitory events. Akt protein kinase B, GSK-3β glycogen synthase kinase-3β, GS glycogen synthase, PYGL glycogen phosphorylase L, FOXO1 forkhead box O1, PEPCK phosphoenolpyruvate carboxykinase, G6Pase glucose 6-phosphatase, Plin2 perilipin 2, SREBP1 sterol-regulatory element-binding protein 1, LD lipid droplet, DGAT2 diacylglycerol acyltransferase 2, TG triglyceride, TLR2 Toll-like receptor 2, NF-κB nuclear factor κB, IL-6 interleukin-6, OSM tumor suppressor m, PGE2 prostaglandin E2, STAT3 signal transducer and activator of transcription 3, SOCS-3 suppressor of cytokine signaling-3, ERK extracellular regulated MAP kinase, IRS-1 insulin receptor substrate-1, OMV outer membrane vesicle, FFA free fatty acid

Since insulin is secreted by β-cells and enters the portal vein, it first reaches the liver, binding to surface receptors on hepatocytes. Then, it triggers the tyrosine phosphorylation-induced activation of insulin receptor substrates (IRSs) and the downstream protein kinase B (Akt). Activated Akt is a signaling hub that regulates downstream glucose and lipid metabolism pathways. However, it is not clear whether periodontitis-derived virulence factors can affect the number and function of insulin receptors on the surface of hepatocytes. It is certain that these virulence factors can affect the expression of proteins that are essential for the aforementioned pathways, resulting in abnormal glucose metabolism, including decreased glycogen synthesis and increased gluconeogenesis (Fig. 4a), as well as steatosis, which is caused by ectopic deposition of free fatty acids (FFAs) (Fig. 4b); thus, these virulence factors contribute to liver IR.

Studies have confirmed that OMVs that are produced at the site of periodontitis can travel from the oral cavity to the liver and enter hepatocytes by endocytosis.^28^ The virulence factors in OMVs can decrease the phosphorylation of insulin receptor substrate-1 (IRS-1), inhibit Akt activity and then regulate glucose metabolism in three ways. First, they can inhibit the expression of glycogen synthase kinase-3β (GSK-3β)and glycogen synthase (GS), thus reducing glycogen synthesis.^29^ Second, they can increase the mRNA expression of glycogen phosphorylase L(*PYGL*), which is a key enzyme in glycogen degradation, and cause increased glycogen decomposition, leading to a decrease in total glycogen synthesis.^29^ In addition, some researchers have confirmed that inhibition of Akt phosphorylation leads to *FOXO1* nuclear translocation,^80^ resulting in increased expression of the gluconeogenic genes phosphoenolpyruvate carboxykinase (PEPCK) and glucose 6-phosphatase (G6Pase)^82^ and leading to increased gluconeogenesis. These changes in these signaling pathways disrupt the maintenance of blood glucose homeostasis, in which hepatocytes participate.

In terms of lipid metabolism, periodontitis-derived virulence factors can upregulate the transcription of the adipose differentiation-related protein perilipin 2 (*Plin2*).^83^ The increased expression of *Plin2* promotes the formation of lipid droplets (LD) and the accumulation of triglyceride (TG) in hepatocytes by activating sterol-regulatory element-binding protein 1 (SREBP1) and the key enzyme of TG synthesis, namely, diacylglycerol acyltransferase 2 (DGAT2).^84,85^ In addition, periodontitis-derived virulence factors activate the TLR2-NF-κB pathway in hepatocytes to promote ectopic FFA deposition^34^ and liver steatosis.^86^ FFAs can upregulate the expression of TLR2 via a feedback loop, resulting in increased sensitivity of hepatocytes to virulence factors and exacerbating liver IR.^87^ In addition, periodontitis-derived virulence factors may also increase the intake of fatty acids by upregulating the expression of the fatty acid receptor CD36 on the surface of hepatocytes, leading to FFA deposition.^88^

In addition to directly affecting hepatocytes to cause IR, periodontitis-derived virulence factors may also regulate Kupffer cells in the liver to induce IR (Fig. 4c). Circulating periodontitis-derived virulence factors can accumulate in Kupffer cells^28^ and activate them.^89,90^ Activated Kupffer cells can secrete IL-6, tumor suppressor m (OSM) and prostaglandin E2 (PGE2), which are important cytokines that induce hepatocyte IR,^91^ IL-6,^92^ and OSM^93^ can induce the phosphorylation and activation of STAT3 in hepatocytes, induce suppressor of cytokine signaling-3 (SOCS-3) transcription and inhibit the phosphorylation of the downstream molecule Akt, while PGE2 inhibits IRS-1 tyrosine phosphorylation by activating ERK1/2.^92^ All of the above effects can inhibit glycogen synthesis in hepatocytes and lead to liver IR. Therefore, the regulatory effect of Kupffer cells on hepatocytes via paracrine inflammatory factors may be another important mechanism by which periodontitis-derived virulence factors regulate liver IR.

In the liver, in addition to the classic insulin signaling pathway, the glucagon signaling pathway also participates in liver IR. Glucagon can bind to the glucagon receptor on the hepatocyte membrane to activate the protein kinase-cAMP system, stimulate PEPCK and G6Pase gene transcription, and promote gluconeogenesis, leading to IR.^94^ Interestingly, periodontitis-derived virulence factors can stimulate the expression of key genes *Ppargc1*, *SIK1* and *Ppp3cc* in the glucagon signaling pathway in the liver.^26^ Therefore, we speculate that the activation of the glucagon signaling pathway may be another important mechanism by which periodontitis-derived virulence factors regulate liver IR, which is worthy of further evaluation in future research.

### Visceral adipose tissue IR

**Fig. 5:**
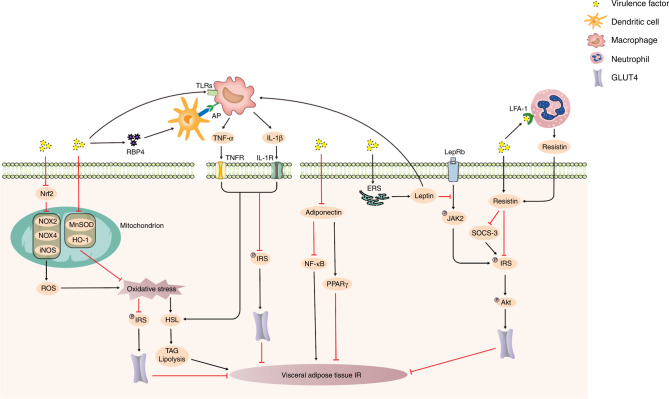
Mechanism underlying visceral adipose tissue IR induced by periodontitis-derived virulence factors. Virulence factors can mediate oxidative stress, activate macrophages to induce inflammatory responses, and disrupt specific adipokine secretion, resulting in visceral adipose tissue IR. Black arrows represent activating events; red arrows represent inhibitory events. TLRs Toll-like receptors, AP antigen presentation, RBP4 retinol-binding protein 4, Nrf2 nuclear factor erythroid-2 related factor 2, NOX2 NADPH oxidase 2, NOX4 NADPH oxidase 4, iNOS inducible nitric oxide synthase, MnSOD manganese superoxide dismutase, HO-1 heme oxygenase-1, ROS reactive oxygen species, IRS insulin receptor substrate, HSL hormone-sensitive lipase, TAG triacylglycerol, TNF-α tumor necrosis factor-α, TNFR tumor necrosis factor receptor, IL-1β interleukin-1β, IL-1R IL-1 receptor, NF-κB nuclear factor kappa-B, PPARγ peroxisome proliferator-activated receptor γ, IR insulin resistance, ERS endoplasmic reticulum stress, LepRb leptin receptor, JAK2 Janus kinase 2, SOCS-3 suppressor of cytokine signaling-3, Akt protein kinase B, LFA-1 lymphocyte function-associated antigen-1, GLUT4 glucose transporter 4

Visceral adipose tissue is also an important insulin-targeting tissue. Insulin stimulates visceral adipocytes to take up glucose and synthesize lipids to maintain blood sugar homeostasis.^95^ However, periodontitis-derived virulence factors can mediate oxidative stress, inflammatory responses, and abnormal secretion of specific adipokines, resulting in impaired insulin signaling, reduced glucose uptake by visceral adipocytes, increased lipolysis, and IR in visceral adipose tissue (Fig. 5).

Studies have shown that LPS can inhibit the activity of the redox transcription factor nuclear factor erythroid-2 related factor 2 (Nrf2),^96^ upregulate the expression of the NADPH oxidase 2 (NOX2), NADPH oxidase 4 (NOX4) and inducible nitric oxide synthase (iNOS) in visceral adipocytes, increase the content of reactive oxygen species (ROS)^97^ and induce oxidative stress by reducing the expression of the antioxidant genes manganese superoxide dismutase (MnSOD) and heme oxygenase-1 (HO-1);^98^ these effects ultimately impair IRS expression^99^ and decrease the activity of the downstream effector glucose transporter 4 (GLUT4),^83,100,101^ resulting in impaired glucose uptake by visceral adipocytes. On the other hand, oxidative stress can also activate hormone-sensitive lipase (HSL), promote the lipolysis of triacylglycerol (TAG),^99,102^ and participate in visceral adipose tissue IR.

In addition to oxidative stress, periodontitis-derived virulence factors also participate in visceral adipose tissue IR through the classical “two-hit” model, namely, the activation of macrophages and the subsequent release of inflammatory factors to induce inflammatory responses. Studies have confirmed that periodontitis -derived virulence factors can stimulate visceral adipocytes to secrete monocyte chemoattractant protein 1,^83,97,103^ induce macrophage infiltration,^104^ and then activate macrophages through TLR pathways. In addition, these virulence factors can also induce the expression of retinol-binding protein 4 (RBP4),^105^ activate dendritic cells, and then activate macrophages via antigen presentation (AP).^106^ Activated macrophages then secrete a large number of inflammatory factors, such as tumor necrosis factor alpha (TNF-α) and IL-1β.^107^ These inflammatory factors can bind to cytokine receptors on cell membranes and reduce the activity of GLUT4 by downregulating the expression of IRS.^108^ These inflammatory factors can also participate in visceral adipose tissue IR by upregulating HSL activity to mediate adipocyte lipolysis to form glycerol.^109^

In addition to inflammatory factors, visceral adipocytes secrete a group of specific adipokines that are involved in the regulation of visceral adipose tissue IR. In response to stimulation with periodontitis-derived virulence factors, specific adipokines, such as adiponectin, leptin, and resistin, are abnormally secreted and participate in IR through autocrine or paracrine mechanisms. Studies have confirmed that adiponectin, which sensitizes tissues to insulin, is downregulated after stimulation with these virulence factors.^76,98^ Decreased expression of adiponectin weakens the inhibition of NF-κB nuclear translocation,^110^ which increases IRS-1 serine phosphorylation mediated by NF-κB to impair the insulin signaling pathway and aggravate IR in visceral adipose tissue.^111^ In addition, adiponectin is an important mediator of insulin sensitivity enhanced by peroxisome proliferator-activated receptor γ (PPARγ). Decreased expression of adiponectin hinders the inhibitory effect of PPARγ on lipolysis, leads to an increased level of FFAs, impairs insulin sensitivity^112^ and promotes IR in visceral adipose tissue. Periodontitis-derived virulence factors can induce endoplasmic reticulum stress (ERS) to promote leptin secretion.^76^ High levels of leptin inhibit the activation of Janus kinase 2 (JAK2) by the cell surface leptin receptor (LepRb) in an autocrine manner and inhibit downstream IRS signaling.^113^ Leptin also promotes the proliferation of macrophages in a paracrine manner, induces the expression of inflammatory factors,^114^ and participates in visceral adipose tissue IR. Resistin is upregulated after exposure to the virulence factors,^97,103^ and it can reduce the phosphorylation of IRS-1 and Akt in visceral adipocytes and reduce GLUT4 membrane translocation and glucose uptake.^115^ It can also inhibit SOCS-3 and impair insulin signaling.^116^ Interestingly, resistin is also released by neutrophils. Leukotoxin can interact with lymphocyte function-associated antigen-1 (LFA-1) on the surface of neutrophils to activate Src family tyrosine kinases, leading to the release of resistin from neutrophil degranulation,^40^ resulting in increased serum resistin levels and therefore participating in visceral adipocyte IR.

In addition to these mechanisms, another important mechanism that is involved in visceral adipose tissue IR is mitochondrial dysfunction. A previous study found that stimulation with periodontitis-derived virulence factors can inhibit the expression of peroxisome proliferator-activated receptor γ coactivator 1α, which is related to mitochondrial biogenesis, in visceral adipocytes,^98^ suggesting that these virulence factors have the potential to mediate mitochondrial dysfunction and promote IR in visceral adipose tissue, which requires further verification in future studies.

### Skeletal muscle IR

Skeletal muscle, similar to visceral adipose tissue, regulates blood glucose homeostasis, and it is responsible for absorbing 80% of postprandial glucose from the circulation; GLUT4 is a key protein that is involved in glucose transport into skeletal muscle.^117^ According to an analysis in a previous study, periodontitis-derived virulence factors mainly cause skeletal muscle IR by activating the immune inflammatory response of the body and disrupting the secretion of adipokines, leading to the abnormal function of GLUT4. Studies have reported that when periodontitis-derived virulence factors enter systemic circulation, they can activate immune cells to secrete inflammatory factors,^40^ leading to increased levels of TNF-α and IL-1β in serum.^118^ These inflammatory factors can reduce the phosphorylation of IRS-1/IRS-2 and Akt, which are key proteins in the insulin signaling pathway, in skeletal muscle,^118^ reduce GLUT4 membrane translocation, and cause glucose uptake disorder and skeletal muscle IR.^119^ Some studies have even observed that this inflammatory effect is transmitted from mother to offspring during pregnancy,^120^ leading to an increase in the phosphorylation of the inflammatory signaling molecules IkappaB kinase α/β, ERK1/2, and NF-κB in the skeletal muscle of the offspring^121^ and thereby downregulating the expression of GLUT4 and inducing skeletal muscle IR.

Abnormal secretion of visceral adipokines in response to periodontitis-derived virulence factors is also an important mechanism that is involved in skeletal muscle IR. As previously described, periodontitis-derived virulence factors inhibit adiponectin secretion from visceral adipocytes,^76^ resulting in a decrease in adiponectin serum levels to 58.7%.^122^ Low levels of adiponectin decrease its interaction with adiponectin receptors on the surface of skeletal muscle, inhibit AMP-activated protein kinase (AMPK) phosphorylation, and reduces downstream membrane translocation of GLUT4.^123^ Under physiological conditions, leptin binds to leptin receptors on the surface of skeletal muscle cells, and this binding initiates an intracellular signaling cascade to promote glucose uptake and glycogen synthesis.^124^ The increase in serum leptin levels caused by periodontitis-derived virulence factors can drive leptin resistance, desensitize leptin receptors and downregulate intracellular signal transduction pathways, and these effects impair glucose metabolism in skeletal muscle.^125^ Similarly, high levels of serum resistin induced by periodontitis-derived virulence factors act on skeletal muscle cells and reduce basic and insulin-stimulated glucose uptake, oxidation and glycogen synthesis, which leads to skeletal muscle IR by altering the function of IRS-1 and Akt^115^ and reducing GLUT4 translocation.^126,127^

As the largest organ system in the human body, the skeletal muscle system accounts for ~40% of the adult body weight,^128^ and the interference of periodontitis-derived virulence factors on glucose metabolism in the skeletal muscle system cannot be ignored. However, compared with liver and visceral adipose tissue, the effects of virulence factors on IR in skeletal muscle are poorly understood. For example, in terms of the mechanism of action, whether periodontitis-derived virulence factors can directly affect skeletal muscle cells to cause skeletal muscle IR, induce the infiltration of immune cells such as macrophages and T cells to indirectly cause skeletal muscle IR, or regulate skeletal muscle IR via both pathways is still unclear. Second, regarding intracellular signaling pathways, the inhibition of the insulin signaling pathway in skeletal muscle cells can lead to impaired glucose transport and glucose phosphorylation as well as reduced glucose oxidation and glycogen synthesis. These are the key mechanisms that lead to skeletal muscle IR.^129^ While previous studies only focused on the effects of periodontitis-derived virulence factors on glucose transport, it will be meaningful to explore the effects of virulence factors on intracellular glucose phosphorylation, glucose oxidation, and glycogen synthesis in the future.

## Effect of periodontal therapy on T2D

Clinically, after periodontal treatment, the count of pathogenic bacteria in subgingival plaque and the titer of IgG antibody in serum against pathogenic bacteria are significantly reduced.^130–133^ Accordingly, the secretion of virulence factors is also decreased, and systemic inflammation is resolved to some extent, as shown by decreased serum levels of the inflammatory factors IL-1β, IL-6 and TNF-α,^134,135^ decreased levels of the adipokines leptin^136,137^ and resistin,^138^ and ameliorated levels of adiponectin.^136^ The serum level of GLP-1 is increased significantly,^139^ which is advantageous for β-cell insulin secretion. The insulin sensitivity of peripheral insulin-targeting tissues is also partially improved, as shown by a reduction in FBG and an improved blood lipid profile.^140^ Additionally, HbA1c, which is a key clinical indicator used to evaluate T2D prognosis, can decrease by 0.43% within 3–4 months after periodontal treatment.^140^ Each 1% reduction in HbA1c can reduce diabetes-related deaths by 21%, myocardial infarction by 14%, and microvascular complications of diabetes by 37%.^141^ The rate of T2D complications and mortality can be decreased if reduced HbA1c levels are maintained for a long duration after periodontal treatment. The improvement in the aforementioned T2D-related indices may be closely related to the effective removal of subgingival pathogenic microflora through periodontal treatment, the elimination of inflammatory tissue decomposition products that provide nutrition for biological disorders, and the establishment of microflora related to periodontal health.^142^

However, it is worth noting that even after periodontal treatment to reconstruct a healthy microbiota, a large number of neutrophils in the patient’s peripheral blood still maintain their proinflammatory status for at least 2 months.^143^ This result may occur because the traditional periodontal treatment mainly focuses on the management of the microbiota without considering the continuous effect of the immune dysfunction caused by periodontal infection. However, the abnormal immune function of the body, as shown by excessive neutrophil inflammation, may increase the risk and severity of T2D. Therefore, it is necessary to develop more comprehensive and effective clinical treatment methods in the future, including the following aspects. First, a new model for microbiota management, such as the application of traditional periodontal therapy combined with laser therapy, antibacterial peptides and probiotics, is needed to quickly and effectively reconstruct a healthy microbiota. Second, treatments that target key virulence factors are needed. For example, the development of specific intervention drugs can accurately and effectively eradicate or neutralize the effect of virulence factors without affecting the surrounding host tissues and commensal microbiota and block their further attack on T2D-related target cells. Third, cell-specific immunotherapy is used to ameliorate the abnormal function of peripheral immune cells caused by periodontitis in order to correct local and systemic immune function disorders and restore metabolic homeostasis.^142^ Finally, target cells must be protected. The development of highly cell-selective drugs can block key pathways through which periodontitis-derived virulence factors damage target cells and thus maintain the normal function of target cells. The combined application of these methods will help to enhance the effect of periodontal treatment, thus providing better treatment strategies for preventing the occurrence and development of T2D and reducing the occurrence of complications.

## Limitations and prospects

In summary, periodontitis-derived virulence factors are involved in the pathological mechanism underlying T2D. Their effects that lead to β-cell dysfunction and mediate IR in peripheral insulin-targeting tissues are being intensively studied. A better understanding of the shortcomings of recent studies will significantly help the future comprehensive elucidation of the impact of periodontitis on T2D and the discovery of new therapeutic targets.

At present, clinical studies have confirmed that microbial dysbiosis in periodontitis is related to the occurrence and development of T2D, and some studies have provided preliminary and effective evidence. However, these studies have only established a relationship between a few bacterial species and the deterioration of blood glucose and IR in small samples, and such studies have only performed mechanistic experiments related to the virulence factors that are secreted by these select bacterial species. In fact, in the dysfunctional subgingival microbiota, in addition to the reported bacteria, there are many bacterial species with significantly increased abundance,^25^ and these bacteria can also synthesize and secrete a large number of characteristic virulence factors. However, at present, the attention given to these new virulence factors is insufficient. For example, little is known about the pathogenicity of these virulence factors and whether they are related to the occurrence and development of T2D. Therefore, future clinical research should carry out large-sample, long-term, multicenter longitudinal observations in a broader population to identify new bacterial species and virulence factors that are related to T2D. By analyzing the longitudinal relationship between these new virulence factors and blood glucose and IR, we can provide a reliable basis for comprehensively exploring the pathological mechanism underlying T2D in the future.

Periodontitis-derived virulence factors are involved in pathological changes in T2D. A single virulence factor does not have a profound effect, but the synergistic effect of many virulence factors can lead to disease. However, only a few virulence factors have been independently studied. In addition, the identities of the virulence factors that play the most important roles in the pathological changes that occur during T2D remain unknown. Thoroughly exploring the synergistic effect of many virulence factors and identifying the key virulence factors involved in pathological changes will help in the development of new therapeutic targets to specifically inhibit the effect of these virulence factors. Combined with existing hypoglycemic agents, these anticipated treatments are expected to improve the clinical therapeutic effects on T2D.

Interestingly, although many studies have reported that periodontitis-derived virulence factors can be detected in β-cells and peripheral insulin-targeting cells and that they can participate in relevant pathological mechanisms, how they are delivered from the periodontal pocket to these cells is unclear. It is essential to identify the carrier and pathway by which these virulence factors are delivered. This information will help in further exploring the mechanism by which these virulence factors escape the immune system in vivo and provide a basis for future immunomodulatory therapies that target these virulence factors. We noticed that the subcellular localization of different virulence factors in target cells was also different. For example, OMVs are located in the cytoplasm,^28^ while gingipain can be found in the nucleus and perinucleus.^27^ Currently, there is no definite answer about whether the differences in virulence factor localization in cells indicate that they mediate different pathological mechanisms, and therefore, further research is needed.

As previously described, periodontitis-derived virulence factors can induce β-cell dysfunction and peripheral IR and thus mediate the occurrence and development of T2D. Recent studies have shown that periodontal bacteria such as *P. gingivalis*, *A. actinomycetemcomitans* and *F. nucleatum* can disrupt the homeostasis of the intestinal microbiota through ectopic colonization^26,144^ and secretion of virulence factors^145^ using mechanisms similar to those in the mouth. This may lead to changes in the abundance of intestinal bacterial species and increase the secretion of virulence factors.^146^ Damage to the insulin signaling pathway mediated by intestine-derived virulence factors is also an important mechanism that leads to T2D. Therefore, we speculate that periodontal pathogens may also promote the development of T2D by inducing the release of virulence factors from intestinal microorganisms, which needs more attention in future research.
